# Lab-on-a-Tip (LOT): Where Nanotechnology Can Revolutionize Fibre Optics

**DOI:** 10.5772/60518

**Published:** 2015-01-01

**Authors:** Qimin Quan, Yiying Zhang

**Affiliations:** 1 Rowland Institute at Harvard University, Cambridge, MA, USA; 2 Geriatric Anesthesia Research Unit, Department of Anesthesia, Critical Care and Pain Medicine, Massachusetts General Hospital and Harvard Medical School, Charlestown, MA, USA

**Keywords:** Lab-on-a-tip (LOT), Nanotechnology, Biosensor, Endoscopy, Single cell analysis

## Abstract

Recently developed lab-on-a-chip technologies integrate multiple traditional assays on a single chip with higher sensitivity, faster assay time, and more streamlined sample operation. We discuss the prospects of the lab-on-a-tip platform, where assays can be integrated on a miniaturized tip for *in situ* and *in vivo* analysis. It will resolve some of the limitations of available lab-on-a-chip platforms and enable next generation multifunctional *in vivo* sensors, as well as analytical techniques at the single cell or even sub-cellular levels.

## 1. Introduction

**Figure 1.**
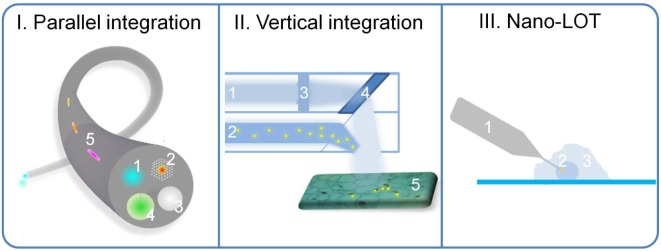
Illustration of integrative LOT devices. I. Parallel integration. An optical fibre LOT that comprises multiple lab functions. Fluorescence detection channel (1), carbon dioxide laser surgery channel (2), white light illumination channel (3), and pressure monitoring channel with a deformable membrane (4) are present on the fibre tip. Other channels, e.g., temperature, pH, oxygen level, etc., can also be integrated along the fibre (5). II. Vertical integration. An endoscope LOT that has two parallel channels: one for endoscopic imaging (1) and the other for molecular diagnostics and therapeutics (2). In the imaging channel, lenses (3) and gratings (or microelectromechanical systems) can be used to steer the light. As suspicious malignant tissues (5) are identified, biologically functional nanosensors (represented by yellow dots) can be injected from the diagnostic/therapeutic channel (2) to perform molecular diagnostics, followed by subsequent *in situ* therapeutic operations (e.g., using gold nanoparticle photothermal therapy). III. Nanoscale LOT device. Nanosensors can be integrated to LOT, and perform analysis at sub-cellular components inside the living cells: e.g., the optical fibre can be fabricated down to a nanoscale tip (1) and nanosensors (2) can be integrated on its tip to perform nucleic acid or protein detection in the nucleus of the cell (3).

Biochemical and immunological assays have been the major analytical methods in biological research, clinical diagnostics, and the drug and food industries. Recently developed lab-on-a-chip (LOC) technologies brought the traditional assays to a higher level of integration, leading to more streamlined processing. They also yielded improved sensitivity and faster assay time by speeding up the chemical reaction and physical processes (thermo-cycling and diffusion) in a confined miniaturized space. As extensive research has already been focused on LOC platforms [1–4], this perspective aims to present the prospects for a complementary platform — lab-on-a-tip (LOT) — that integrates one or several laboratory functions on a single miniaturized tip. Although the assays could be performed by optical, electrical, or mechanical means, we will focus our discussions on optical measurements in this article. Early examples of LOT platforms include fibre optic intracranial pressure sensors, thermometers, and endoscopes. However, LOT devices are at an early stage —devices are typically mono-functional, fabrication methods are not standardized, and most devices are macroscopic. We envision more comprehensive lab functions to be integrated on LOT (Fig. 1). In particular, devices will measure multiple physical and chemical properties (parallel integration), and will integrate imaging, diagnostics, and therapeutic functions (vertical integration). We also anticipate novel and scalable LOT fabrication techniques will be developed and standardized, and further development of nano-LOT devices will enable new research at single cell or sub-cellular levels.

## 2. Multifunctional LOT devices

The great success of fibre optic technology in the telecommunication industry led to the development of their applications in the biomedical and pharmaceutical industries. Optical fibres offer natural “lab-on-a-tip” operation, and were extensively developed during 1980s and 1990s [5]. The fibre optic intracranial pressure sensor is one of the earliest developed LOT devices. It is used in continuously monitoring the intracranial pressure of head trauma patients, and works by sending light to the tip of the fibre where it is reflected back from a diaphragm that deflects with external pressure. Non-optical piezo-resistive or capacitive transducers also exist, but they suffer from the risk of electrical-shock hazard and long-term drifts, while fibre optic intracranial pressure sensors are electrical shock hazard free. They are also small, flexible, and have long-term reliability. First commercialized by Camino Labs in the 1980s, fibre optic intracranial pressure sensors are now widely used in diagnosing potential elevation of intracranial pressure and monitoring its progression in the clinical neurological examination.

Another LOT success is the fibre optic thermometer used in hyperthermia therapy, which is used to kill cancer cells or to make cancer cells more sensitive to radiation therapy by exposing body tissue to a slightly higher temperature. The temperature of the tissue is controlled by the application of microwave or radiofrequency radiation, and must be precisely monitored. Fibre optic thermometers offer the unique advantages of natural electrical isolation and freedom from electromagnetic interference. Different measurement modalities exist [6], the most well-known ones are temperature sensitive fluorescent phosphors and GaAs crystals patterned on the fibre tip that provides spectrum features sensitive to the temperature. It was first commercialized by Luxtron Inc. in the 1980s, and subsequently developed by many other companies.

Fibre optic biosensors were also used to measure physical parameters such as pH, blood flow rate, blood oxygen levels, radiation dosage, and biting force in dentistry. New materials, especially metals and semiconductors have been integrated into glass or polymer fibres to achieve optoelectronic properties [7]. Composite geometries, such as photonic crystal fibres [8] and hollow core Bragg fibres [9], have been developed. They have the advantage of guiding light in their hollow cores, thus allowing high power applications (e.g., laser surgery [10]).

One future development is likely to be the multifunctional fibre optic sensors. An interesting analogy can be made between fibre sensors in the medical industry and the drills in the oil and gas industry. A large number of sensors are equipped along the drill and perform comprehensive measurement while drilling, including depth, pressure, gas composition, resistivity, porosity, gamma ray, drilling system orientation, and wellbore geometry [11]. We envision more functions will be integrated into fibre optics and catheters, and thus allowing for more powerful and comprehensive monitoring during diagnostic or therapeutic operations. Since fibre optic sensors have much smaller dimensions than drills, more powerful and scalable techniques will need to be developed. Nanofabrication techniques are already well developed for planarized surfaces (such as chips and wafers), but much less so for non-traditional surfaces. Several strategies are currently being explored, including post-transfer chips onto fibres [12, 13], and direct fabrication on fibre facets by focused ion beam (FIB) [14] and electron beam lithography (ebeam) [15]. Traditional fibre drawing techniques are also extended to new materials, including metals, semiconductors, and polymers [16].

## 3. Next generation of endoscopy

Endoscopy allows diagnostics inside the human body by bringing an optical system in proximity to the tissues of interest. It also revolutionized surgery, allowing minimally invasive procedures to be performed inside the body. Philipp Bozzini (1773–1809) was credited with the invention of the first endoscope about 200 years ago, and Max Nitze (1849–1906) was among the first to take endoscopic operations inside the bladder. Since Basil Hirschowitz (1925–2013) developed the first fibre optic endoscope, many improvements were made, including using chargecoupled devices (CCD) for imaging and rod-lens systems to improve the quality of imaging. In 1997, optical coherence tomography (OCT) endoscopy demonstrated improvement of imaging resolution from the previous 100 um to 10 um with large penetration depth [17]. Subsequently, two-photon and multi-photon imaging (MPM) [18, 19] further improved the imaging resolution (∼3 um), but they have inferior penetration depth compared to OCT in general. Optical frequency-domain imaging (OFDI) [20] and more recently Doppler OFDI [21] improved the imaging speed by several orders of magnitude over OCT. Its deep penetration made it possible to image significantly larger tumour volume than MPM. Furthermore, scanning fibre wide-field fluorescence imaging [22] provides information about fluorescent labelled molecules or autofluorescent proteins. Coherent Raman scanning fibre endoscopy [23] provides some chemical information, such as CH_2_ or CH_3_ bonds, without labels. Efforts to combine multiple imaging modalities that reveal both morphology and molecular information are currently being actively pursued. For example, recently developed OCT and fluorescent imaging [24], OFDI and near-infrared fluorescence imaging [25], provide both imaging and molecular information.

For more than a hundred years, endoscopists have relied on the visualization of the morphology of tissue with subsequent biopsy procedure for immunological and molecular diagnostics. The next generation endoscopy will extend its capability from imaging to diagnostics and therapy, which we call vertical integration. Fluorescent labelling [24, 25] or other label-free biosensors can be integrated to the endoscope and bring immunological and molecular diagnostics *in situ.* Therapeutic capabilities can be integrated as well. For example, gold nanoparticle photothermal therapy with near-infrared light [26] or visible laser [27] has been demonstrated in mice model. However, more controlled methods to provide irradiation and alternative methods to enhance the *in vivo* delivery are required [28]. The next generation endoscopy will integrate high-resolution three-dimensional imaging, immunological and molecular diagnostics, and subsequently photothermal therapy with targeted delivery of nanoparticles on the malignant cells.

## 4. Nano-LOT devices for single cell analysis

Most of our knowledge in biology so far has been based on ensemble measurements on cells. Patch clamp technology made it possible to record the currents of single ion channels for the first time [29], for which Erwin Neher and Bert Sakmann shared the Nobel Prize in Physiology or Medicine in 1991. More recent development of nanoscale patch clamp techniques made it possible to record single ion channels at specific locations such as the presynaptic terminal, when used in combination with super-resolution (below optical diffraction limit) imaging techniques [30]. Electrophysiology measurement is also achieved with less invasive systems, such as Pt nanowires pulled in glass capillaries [31], carbon coated nanopipes [32], and freestanding kinked nanowire transistor nanoprobes [33].

Other physical and chemical parameters of single cells can also be measured with optical methods. In 1992, Tan et al. fabricated a sub-micron fibre tip which was coated with pH-sensitive fluorescent polymers to detect pH levels in a rat embryo [34]. Vo-Dinh et al. further miniaturized the tip and demonstrated detection of fluorescent molecules inside single cells [35, 36]. Calcium level [37] and lactate concentration [38] measurements were also demonstrated by using fluorescent dyes coated on the fibre nanotips. Raman spectra were measured inside single cells by coating gold nanoparticles on the glass capillary tip in order to enhance the Raman signals [39]. Recently, Hong et al. measured the dynamics of intracellular p53 proteins using a nano-fibre tip coated with a single gold nanoparticle [40], extending its capability to detect non-fluorescent proteins in single live cells. Other materials, for example carbon nanotubes, have been used to deliver quantum dots into cells [41], as well as to study the force and indentation depth during cell penetration [42]. Boron nitride nanotubes were also used to deliver quantum dots [43]. When the process of inserting these nanotips into cells was studied using confocal imaging [44], it was found that the dimension of the tip is of critical importance: sub-100 nm dimension will not damage the cell membrane or disturb the cellular energetic system [45]. Nano-LOT devices offer opportunities to study single cells and their biochemical compositions at high spatial resolution, as well as their temporal response to external stimuli in their natural living conditions [46]-[48]. We envision that versatile nano-LOT devices will be proposed and developed, and that more delicate structures and sophisticated mechanisms will be invented for precise monitoring and controlling at the single cell level.

## 5. Summary

We reviewed the early development of LOT devices, including fibre optic sensors, endoscopes, and more recently developed nanoscale bioprobes. With the rapid advance of nanotechnology, LOT devices will become multi-functional, will integrate imaging, diagnostics, and therapeutic capabilities, and will become more powerful in analysing smaller biological components such as single cells. LOT platforms will be a great complement to LOC platforms, and together they will offer comprehensive analysis and control of biological systems both *in vitro* and *in vivo*.
